# Cases of Bronchocele Cured or Relieved by Iodine

**Published:** 1823-08

**Authors:** Wm. Rickwood


ART. VI.—*Cases of Bronchocele cured or relieved by Iodine.* By WM. RICKWOOD, jun. Esq.

CASE I.—Sarah Geal, of Slinfold, aged twenty-seven, was first affected with enlargement of the thyroid gland about the age of puberty, and it became so large as to occupy the angle between the jaws; it protruded beyond the chin, and the lower part rested on the chest. She breathed and swallowed with great difficulty, and was frequently unable to lie down in bed. She began to take the tincture of iodine,* in the quantity of ten minims twice a-day, on the 24th of April, 1821: by the 1st of May, the tumor was much reduced in size; and, by the end of June, it was not larger than a pigeon's egg, chiefly consisting of the loose integuments of the throat. She took, during the whole time, nearly three ounces of the tincture.

The second case was that of —— Street, aged seventeen. The bronchocele had commenced about two years previously, and had acquired a considerable size: the right lobe of the gland was the largest, and which I have uniformly observed in every case that I have met with in this neighbourhood. This patient began to take the tincture of iodine on the 25th of September, 1821, and continued it regularly until the 14th of January, 1822; by which time the swelling had *nearly* disappeared.

CASE III.—A young lady, aged nineteen, applied to me with an enlargement of the lower portion of the thyroid gland. It did not protrude much outwardly; neither was it pendulous, as in the two former cases; but it felt hard to the touch. This patient began the use of the iodine (the tincture,) on the 14th of July, 1822, increasing the dose from ten to fifteen drops twice in the day, until the 14th of October, but without the least alteration in the size of the tumor: it did not, however, *increase*, which it was continuing rapidly to do before the iodine was employed.

CASE IV.—Mrs. A. aged forty-nine. In this patient the

---

* Iodine, gr. xl.; Alcohol, ℥j. M.

bronchocele had existed, without much inconvenience, for above twenty years: latterly, it had increased considerably; and, from the pressure made on the trachea and œsophagus, she both breathed and swallowed with great difficulty, giving her at times the feeling of suffocation; there is also considerable pain at times in the gland. Eight leeches were applied to the part, which bled freely, and relieved the pain. On the 4th of January, 1823, this lady commenced taking the iodine, and continued it, with occasional intermissions, until the 14th of March; by which time the tumor was considerably lessened, and was no longer troublesome. This is the only case in which the iodine produced any unpleasant effects; the patient complaining of its giving her occasionally pain in the stomach and head, which subsided immediately upon discontinuing it.

I have three additional cases now under treatment, all of whom are improving. The two first cases above related had formerly used the burnt sponge, with temporary benefit; they now remain quite well. Upon the whole, I feel perfectly convinced that the iodine is, in this disease, more certain in its operation than the sponge; and I have also found it to be an excellent tonic in many other complaints.

*Horsham, Sussex; May* 1823.

